# Use of a new antimicrobial consumption monitoring system (Vet-AMNet): Application to Dutch dairy sector over a 9-year period

**DOI:** 10.3389/fvets.2022.984771

**Published:** 2022-12-15

**Authors:** Pedro Moura, Pim Sanders, Dick Heederik, Ingeborg Marianne Van Geijlswijk, João Niza-Ribeiro

**Affiliations:** ^1^Instituto de Ciências Biomédicas Abel Salazar, Universidade do Porto, Porto, Portugal; ^2^The Netherlands Veterinary Medicines Institute (SDa), Utrecht, Netherlands; ^3^Institute for Risk Assessment Studies, Faculty of Veterinary Medicine, Utrecht University, Utrecht, Netherlands; ^4^Division of Veterinary Pharmacotherapy and Pharmacy, Department of Population Health Sciences, Faculty of Veterinary Medicine, Utrecht University, Utrecht, Netherlands; ^5^Laboratório associado para a Investigação Integrativa e Translacional em Saúde Populacional (ITR), Porto, Portugal; ^6^Epidemiology Research Unit (EPIUnit), Instituto de Saúde Pública da Universidade do Porto, Porto, Portugal

**Keywords:** antimicrobial, consumption, surveillance, stewardship, resistance, dairy (cows), monitoring

## Abstract

**Introduction:**

The urgency of preventing the increase of antimicrobial resistance has been emphasized by international authorities such as the World Health Organization, European Medicines Agency, and World Organization for Animal Health. Monitoring systems capable of reporting antimicrobial consumption data are regarded as a crucial pillar of this fight. The Vet-AMNet system was developed to collect and analyze national antimicrobial consumption data in Portuguese dairy farms to support the veterinary authority in stewardship actions and to assist both veterinarians and farmers in daily decisions related to antimicrobials.

**Methods:**

To evaluate the robustness of the system and other identified critical success factors, it was used to analyze antimicrobial consumption data available from the Dutch dairy cow sector over the period from 2012 to 2020. The data previously used for publications by the Netherlands Veterinary Medicines Institute (SDa) were imported and pre-processed by the Vet-AMNet system according to the SDa's standard operating procedure and the Dutch metrics to measure antimicrobial consumption were calculated.

**Results:**

By comparing the outputs with the figures generated by the system established in the Netherlands, the Portuguese system was validated. Antimicrobial consumption data from the Dutch dairy sector during the 9-year period will be presented in unpublished graphs and tables, where each molecule's pharmaceutical formulation, pharmacotherapeutic group and line of choice will be related and discussed, illustrating the evolution of sectorial antimicrobial consumption against a background of a strong national antimicrobial policy initiated by public-private cooperation and supported by legislation.

## 1. Introduction

To understand and control the emergence of antimicrobial resistance (AMR), it is essential to monitor the use of antimicrobials and resistance development (1). The main aim of veterinary antimicrobial consumption (AMC) surveillance programs is the promotion of prudent use of these substances, among veterinarians and farmers, which can be achieved by interpreting patterns and tendencies of use related to the emergence of AMR (2). In the Netherlands, the association between the reduction of AMC and a decrease in the prevalence of AMR genes in indicator *Escherichia coli* has been proven, in several livestock sectors (3).

AMC monitoring systems establish a foundation for evaluating the effectiveness of implemented control measures, by identifying the emergent use of certain antimicrobial (AM) substances, enabling risk evaluation and management. They also allow the comparison of AM usage at a national and international level, when the same indicators are applied, and within a given time frame (2).

The European Medicines Agency (EMA) started the European Surveillance of Veterinary Antimicrobial Consumption (ESVAC) project, with the purpose of collecting data on AMC in animals to inform policy makers. National participation has been established on a voluntary basis. However, from 2023, reporting AM sales and consumption to EMA will be a legal obligation, after Regulation (EU) 2019/06 on veterinary medicinal products (4).

The Vet-AMNet project started in 2019, as a conjoint initiative of the *Instituto de Ciências Biomédicas Abel Salazar*—Porto University (ICBAS-UP), the Portuguese Veterinary Authority (DGAV) and the Portuguese Dairy Farmers Association (ANABLE). The system's main goal is to collect and analyze veterinary AMC data within Portuguese dairy farms. It will be used by DGAV in the context of acquiring information for the ESVAC project, and to respond, by January 2023, to the AMC data submission requirements stated in Regulation (EU) 2019/06. Besides this main goal, it has also been created with the aim of sharing mobile dashboards where AMC figures are presented along with associated costs, milk yield values and information captured *via* field questionnaire. Therefore, it may be used by veterinarians and farmers in their daily activities as an analysis tool to assist in AMC related decision making.

The following characteristics were identified, at this stage, as critical success factors to the systems' good performance and longevity:

- Flexibility: to allow adaptations to different output requirements and future data processing alterations.
- Universality: it should be compatible with most data input formats, so future collaborations are not restricted by input norms.
- Real time responsiveness: after establishing the system's architecture, it should be able to produce outputs in a time-efficient way, after the submission of predesigned standardized inputs.
- Customizability: as the need to guide decisions in an efficient way was identified, the system was designed to be able to provide different stakeholders with information that is relevant to their needs.

In what concerns veterinary antimicrobial consumption, the Netherlands is considered to be an international success model, where the combination between voluntary and mandatory actions (5) has led to an overall reduction of 70.8% in kilograms of antimicrobials sold in the Dutch livestock sector, since 2009. Antimicrobial usage in the Dutch dairy sector is considered to be low and acceptable by the livestock sector and by the Netherlands Veterinary Medicines Institute (SDa) expert panel, with most of the usage relating to individual animal treatments, and a very low consumption of substances of critical importance (6).

The SDa aims to promote the responsible use of antimicrobials (6), protecting public health while taking animal welfare into account. The institute receives the totality of the AMC data annually. Dutch livestock sectors receive AMC data reports from the SDa, but have their own dynamics regarding data analysis and communication with farmers and veterinarians (7). The SDa, by publishing annual reports, provides insight to the government and general public regarding national AMC figures, and establishes regularly updated consumption targets (benchmark values) (6).

This work aims to:

- Validate the Vet-AMNet system, by assessing its robustness to sustain a country level AMC monitoring program and evaluate the critical success factors identified, by recreating the data analysis produced by the AMC monitoring system that is implemented in the Netherlands and has been used to produce previously published reports.
- Provide guidance for the design of an AMC monitoring system.
- Assess the detailed consumption of AM substances in Dutch dairy farms by differentiating the sectorial figures into pharmacotherapeutic groups, pharmaceutical formulation, national line of treatment and segmenting farms into percentiles according to their AMC. The previously unpublished outputs generated, by correlating the mentioned variables, demonstrate new perspectives on the evolution of the sectorial AMC. These will be interpreted in the scope of the likely effect of restriction policies and other measures implemented, over this period, to promote a more responsible use of AM substances.

## 2. Materials and methods

### 2.1. Antimicrobial drug information

Information regarding the dosages of veterinary antibiotic drugs registered in the Netherlands is contained in a database called the “DG-standard.” In this, each drug is associated with a specific “number of treated animal kilograms” which corresponds to the number of animal biomass kilograms that may be treated by using one package of the specific drug in question. The “DG-standard” database also encompasses the European Article Number (EAN), pharmacotherapeutic group, pharmaceutical formulation, package units and size for every licensed veterinary antimicrobial product since 2003 (1). Antimicrobial molecules for veterinary use have also been classified into first, second and third line of choice for animal treatments, according to national treatment guidelines published by the Veterinary Antimicrobial Policy Working Group (WVAB) of the Royal Dutch Veterinary Association (KNMvD), based on directives form Dutch Health Council (2, 3) In Supplementary Table 1, the pharmacotherapeutic groups of the veterinary antimicrobial medicines registered for use in dairy cattle can be seen, together with their line of choice classification.

### 2.2. Dutch antimicrobial usage indicators

To express the amount of antibiotics used in the Netherlands within a particular livestock sector, the SDa has developed two indicators that correspond to the defined daily doses animal (DDDA) consumed in a given year, the DDDA_NAT_ and the DDDA_F_ (1).

The DDDA_NAT_ is used to evaluate trends in AMC at a national level and represents the average number of days/year an average animal, within a particular livestock sector, is treated with AM. It is calculated by dividing the number of treated animal kilograms times the number of days within a livestock sector for a particular year by the average number of total animal kilograms present within the livestock sector concerned, for the given year (1), as shown in Formula 1 in the Supplementary material.

The DDDA_F_ is used to assess AMC at farm level and compare a farms' consumption with a predefined benchmark value. It represents the number of days per year an average animal is treated, at that farm. It is calculated by dividing the number of treated kilograms times the number of days on a farm for a particular year by the average number of kilograms of animals present on that farm (1), as shown in Formula 2 in the Supplementary material.

The use of the national defined daily dose animal (DDDA_NAT_) units allows the standardization of country level AMC and its comparison over time by categories of choice, pharmaceutical formulation, and individual molecules. Unlike the DDDA_F_ this method is not influenced by redefinition of population parameters, as it happened for instance in the pig sector, when the combined population of sow and piglets was split in two distinct populations, sows + suckling piglets and weaned piglets. Therefore, the DDD_NAT_ can be used to follow trends in AMC over time and to assess the impact of interventions within a livestock sector. Also, the size of the farm is not influencing the outcome since all AMC is related to all animals within a livestock sector in the country.

The consumption of individual farms, expressed in DDDA_F_, allows the analysis of the differences between farms, and may be helpful in identifying parameters influencing AMC. This indicator is also used to benchmark the farm's AMC. It is important to realize that in the national overall average farms' defined daily dose animal (DDDA_F_), small farms and big farms have identical impact, while the DDDA_NAT_ reflects the weighted average of DDDA_F_. This indicator also allows the assessment of the impact of interventive actions at farm level. Some farms may register 0 DDDA_F_, over a year. This can happen if a farm identification number is associated with registered dairy cattle, but with no antimicrobial prescriptions over the given period.

### 2.3. Policy measures introduced and market fluctuations in the Netherlands since 2012

To facilitate the interpretation of the findings in AMC in dairy cattle, we summarize the most relevant policy measures introduced and market fluctuations in the Netherlands since 2012, in Table 1.

**Table 1:** Relevant policy and market fluctuations restricting the use of antimicrobials in the dairy sector in the Netherlands since 2012.

**Year**	**Policy/market fluctuation**	**Main effect**	**Source**
2009	Establishment of General reduction targets for the use of antibiotics in livestock set by Dutch government:	A reduction in antibiotic use across all monitored livestock sectors.	SDa note on precision reduction targets (8)
	−20% reduction in overall in antibiotic use by 2011;		
	−50% reduction in overall antibiotic use in 2013.		
Before 2012	Lack of products with first line AM substances for treating mastitis, and limited popularity of these to be used in dry cow therapy	Mainly second line intramammary (imm) products were used, both in dry cow and lactating cow treatment	2012–2013 SDa report
2013	Royal Dutch Society for Veterinary Medicine (KNMvD) published its guideline on the application of selective dry-cow therapy (“Selectief droogzetten”)	Decrease on the overall usage of dry-cow imm products overall and a shift from products with combinations of antibiotics toward products with first line penicillins	2014 SDa report
2013	Introduction of more strict legislation allowing only first-line AMs for individual treatment to be available in small amounts for the farmer	Important reduction of AM's stock at farms	Regulation of the state secretary for economic affairs of 15 August 2013, no. WJZ/13031524 (9)
2017	Introduction of first-line mastitis injectors in NL	Shift from lactating cow second-line products to first-line imm products	2017 SDa report

### 2.4. Data sources

To calculate the DDDA (DDDA_F_ and DDDA_NAT_) values, the SDa is supplied by the livestock sectors with information, at farm level, regarding the average number of animals present and veterinarian antimicrobials prescription registries.

In the Netherlands, veterinarians working in the veal, broiler, turkey, cattle, pig, rabbit and goat farming sectors are obliged to report all AM prescriptions into the livestock sector database, which is mostly done *via* software packages with a Practice Management System (PMS) like Animana^®^, Easyvet^®^ or VIVA^®^. These PMS's are in place to register all interventions and dispensed medication to clients e.g., farms by veterinarians for veterinary care, logistic and financial purposes. The usage data of antimicrobial medication are then provided to the SDa by the sector quality systems, supplemented with the animal data. Data quality regarding AM prescriptions is ensured by requirements that the SDa has established and can be consulted in the standard operating procedure of the organization (4).

Records from 2012 to 2020 containing farm-level prescriptions, animal population and relevant drug characteristics, such as the Dutch national defined dose-based unit of measurement “DDDA,” active ingredient, substance group, and pharmacotherapeutic formulation, as previously used for publications by the Netherlands Veterinary Medicines Institute (SDa) were imported in and analyzed by the Vet-AMNet system. The analyzed dataset comprises all the Dutch farms that registered at least one dairy cow in the covered years, encompassing a yearly average of around 17,000 farms and 495,000 treatment registries.

The animal sector quality systems also provide the average number of animals present over the period of a given year and these figures are either collected by inspection visits or extracted from the national mandatory “Identification and Registration System (I&R)” for animal registration (4).

To assess the average number of live animal kilograms on a farm, which represents the animal population at risk of being treated with antimicrobials, a standardization of the animal biomass denominator had to be made, to make the estimates feasible and more precise. Therefore, in the case of dairy cattle herds, animals are split into 4 categories, with divisions related to age. Each of these categories is associated with a previously estimated and defined standardized weight per animal (1). The analyzed dataset amounts for a yearly average of around 1.6 million animals and the animal categories and associated weights can be found in Supplementary Table 2.

### 2.5. Data analysis using the Vet-AMNet system

All the data gathered was systematically pre-processed using the Vet-AMNet system; mainly done by aggregating entries from the original source and by shaping data to the desired structure, designed to harmonize all inputs. Herd data inputs were aggregated by farm and year and then multiplied by the standard weight values defined by the SDa. AMC inputs were aggregated by year, farm, active ingredient, and pharmaceutical formulation. Drugs without defined antimicrobial use in cattle in DG-Standard were removed from the analysis.

**Figure 1:**
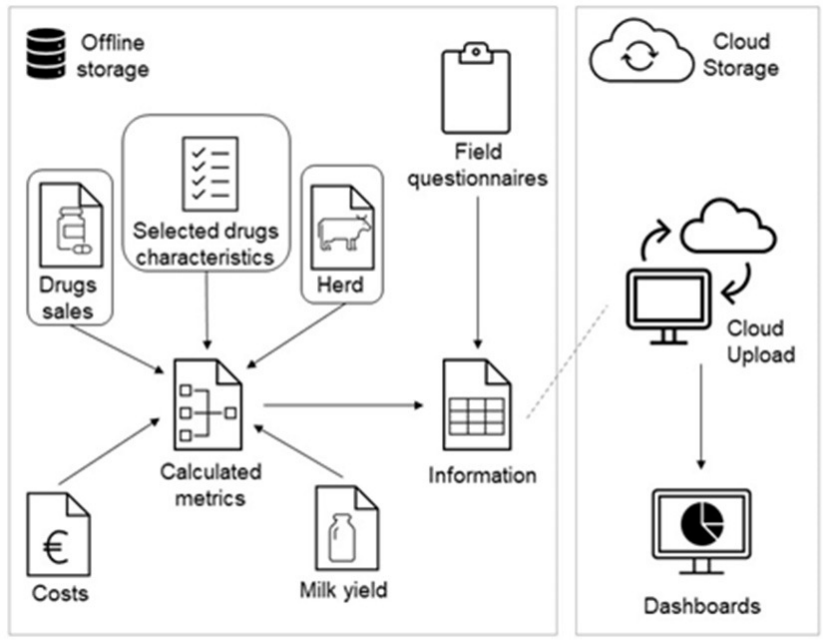
Vet-AMNet's adapted data architecture. The metrics calculated in this analysis required “drug sales,” “herd data,” and “selected drugs characteristics” as highlighted in the model.

After pre-processing all data inputs, these were modeled into an adapted version of the Vet-AMNet data architecture, illustrated in Figure 1. Components including milk yield data provided by the dairy cooperatives and the costs of antimicrobial drugs are also part of the original Vet-AMNet system, together with farm assessment questionnaires collected by veterinarians on topics such as biosecurity. However, these were not encompassed in the present analysis since the main aim of this work was to validate the use of the Vet-AMNet system to report national AMC monitoring figures alone.

The European article number (EAN) was used to connect the drugs prescribed with the specified product characteristics present in the official list of licensed AM drugs (DG-standard), and each farm's AM sales data was related to its respective animal data based on each farm's unique identification number. The indicators described above (DDDA_F_ and DDDA_NAT_) were calculated, per each of the analyzed years, also using the Vet-AMNet system. The components of the Vet-AMNet system that were used in this analysis were built on Microsoft Power BI Version: 2.103.881.0.

## 3. Results

### 3.1. Validation of the Vet-AMNet system

The Dutch indicators used to measure AMC were recreated using the same source data and achieving the same figures as previously generated and published by the SDa, in their yearly reports on the usage of antibiotics in agricultural livestock in the Netherlands (5–14). A compilation of these figures published by the SDa may also be found in Supplementary Table 3, by comparison, it can be verified that the figures presented in the graphs and tables produced by the Vet-AMNet system and presented in the next sections are accurate recreations of the overall figures produced by the Dutch system.

The information produced by the Vet-AMNet system, regarding the last 9 years of AMC in the Dutch dairy sector, was compiled and converted into dashboards composed of different visuals such as the tables and graphs presented in this segment, that were developed to meet the SDa's expert panel data visualization requirements.

### 3.2. Overall AMC in the Dutch dairy sector

**Figure 2:**
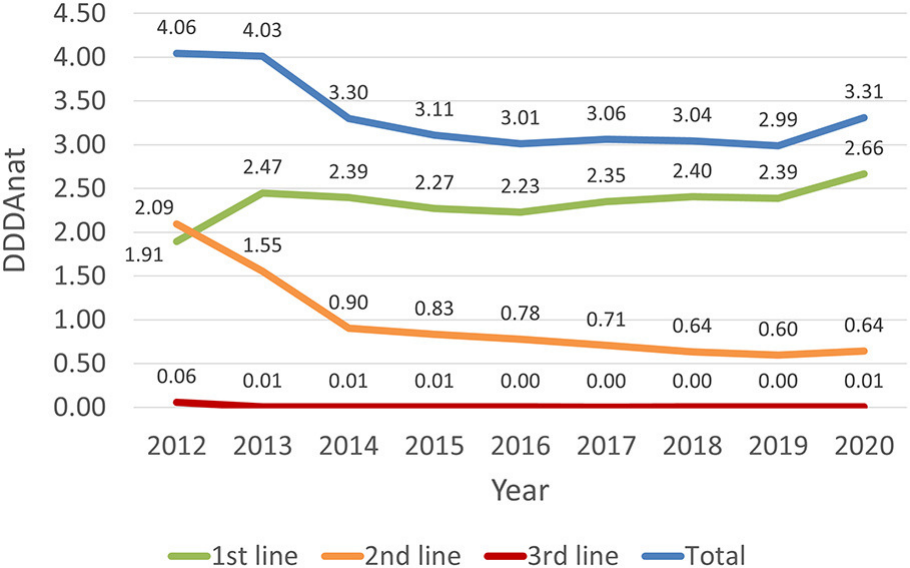
Antimicrobial consumption in the Dutch dairy sector, in DDDANAT units, from 2012 to 2020, divided in total, 1st, 2nd and 3rd line products.

AMC in the Dutch dairy cattle sector can be considered low over the entire study period. An average cow received antimicrobial treatment for <5 days per year (5 DDDA_NAT_) in all the years AMC was recorded. As depicted in Figure 2, the overall consumption of antimicrobial substances, between 2012 and 2013, was similar. From 2013 to 2014, there was a 24% decrease in consumption, and a further 6% reduction from 2014 to 2015. The sector then achieved equilibrium from 2016 to 2019, registering a consistently low yearly consumption of ~3 DDDA_NAT_, with the SDa expert panel reporting that these small percentual variations were expected and not a concern (5). From 2019 to 2020 overall AMC increased 11%.

Figure 2 shows that the overall reduction in AMC was mainly due to a marked decline in the use of second-line products between 2012 and 2014 (−57%) which then continued until 2019, although in a much less abrupt way. In the 9 years covered, the use of second line AM reduced from 2.09 to 0.64 DDDA_NAT_, representing an overall reduction of ~69%. The use of third-line products was already residual since 2013, and therefore fluctuations in the use of these substances did not have a notable impact in the overall consumption. In contrast, the use of first line AM products grew from 1.91 to 2.45 DDDA_NAT_, increasing almost 23%, from 2012 to 2013, resulting from a shift from second-line products. After this increase, the use of these molecules remained almost constant, in the 6 years that followed, and increased by 11% from 2019 to 2020.

In 2012, the first year of full coverage AMC monitoring in the dairy cattle sector, first line antimicrobials were not the most used products, accounting for 47% of the treatments. They became the most used line in the following year, with a 29% increase in the consumption of these substances at the cost of second line products, which can be seen in Table 2. In 2014, the consumption of first line products stabilized, representing over 70% of the treatments, with a steady relative growth each year and reaching more than 80% in the 2020. The relative consumption of second line products decreased sharply from 2012 to 2014, and gradually from 2014 to 2020, representing less than 20% of the usage registered in 2020. The consumption of third line products decreased from 2012 to 2013 and represents a minor fraction of total AMC.

**Table 2:** Yearly antimicrobial consumption, in DDDA_NAT_ units, from 2012 to 2020 by pharmaceutical form and by line, in consumption and overall variation (Total Var).

**Pharmaceutical forms**	**2012**	**2013**	**2014**	**2015**	**2016**	**2017**	**2018**	**2019**	**2020**	**Average**	**Total Var**
Imm dry	1.87	1.97	1.40	1.29	1.30	1.3	1.30	1.24	1.33	1.45	−29%
1st line	0.98	1.41	1.36	1.26	1.26	1.3	1.28	1.22	1.30	1.26	33%
2nd line	0.89	0.56	0.04	0.03	0.03	0.0	0.03	0.03	0.03	0.19	−97%
3rd line	0.00	x	x	x	x	x	x	x	x	x	x
Imm lactating	0.79	0.82	0.76	0.71	0.65	0.7	0.70	0.70	0.80	0.74	2%
1st line	0.00	0.01	0.01	0.01	0.00	0.2	0.21	0.25	0.34	0.11	8300%
2nd line	0.77	0.81	0.75	0.70	0.64	0.6	0.49	0.45	0.47	0.63	−39%
3rd line	0.02	0.00	0.00	0.00	0.00	x	x	x	x	x	x
Intra-uterine	0.15	0.14	0.13	0.11	0.10	0.1	0.09	0.08	0.08	0.11	−47%
1st line	0.12	0.12	0.11	0.10	0.09	0.1	0.08	0.07	0.07	0.09	−41%
2nd line	0.03	0.03	0.02	0.02	0.01	0.0	0.01	0.01	0.01	0.02	−68%
Oral	0.11	0.06	0.04	0.04	0.04	0.0	0.03	0.02	0.02	0.04	−78%
1st line	0.05	0.05	0.04	0.03	0.02	0.0	0.02	0.01	0.01	0.03	−71%
2nd line	0.06	0.02	0.01	0.01	0.01	0.0	0.01	0.01	0.01	0.02	−84%
Parenteral	1.13	1.02	0.97	0.96	0.94	0.9	0.93	0.95	1.07	0.98	−5%
1st line	0.74	0.87	0.89	0.88	0.85	0.8	0.83	0.84	0.94	0.85	27%
2nd line	0.35	0.14	0.08	0.08	0.08	0.1	0.10	0.10	0.13	0.13	−64%
3rd line	0.04	0.01	0.00	0.00	0.00	0.0	0.00	0.00	0.01	0.01	−86%

### 3.3. Farm level AMC in the Dutch dairy sector

In Table 3, the average consumption pattern of the sector farms (DDDA_F_) is split in quartiles, and the range between them and their respective yearly tendencies are shown, highlighting the response of the sector segmented into different AMC categories. On average, the interquartile range between the second and third quartiles is 1 DDDA_F_. The average range between the 5th and the 25th percentile is 1.13 and between 75th and the 95th is 1.5, showing a slight right skewness. Average consumption of farms on the 5th percentile remains relatively constant across the whole period. In percentile 25th, no significant move is visible in the second year, but a strong reduction can be seen from 2013 to 2014, with low reductions in median farms and below, until 2019. A general increase is noticeable in 2020. From 2016 onwards, farms from 95th and 75th percentile diverge from the median. A visual representation of the percentiles described in Table 3 may be found in Supplementary Figure 1. The number and proportion of farms that registered zero AMC in the analyzed period remains stable around 2%.

**Table 3:** Total number of farms, average farm's antimicrobial consumption, percentage of zero consumption farms, and percentiles 5, 25, 50, 75 and 95 from 2012 to 2020, in DDDA_F_ units.

**Year**	**No of farms**	**Zero consumption farms**	**Average DDDaf**	**DDDaf percentile 5**	**DDDaf percentile 25**	**Median DDDaf**	**DDDaf percentile 75**	**DDDaf percentile 95**
2012	18,053	394	2.9	0.23	1.63	2.72	3.75	5.6
2013	18,005	296	2.79	0.3	1.78	2.78	3.7	5.29
2014	17,747	229	2.27	0.29	1.37	2.19	3.04	4.47
2015	17,737	227	2.16	0.27	1.29	2.08	2.91	4.24
2016	17,529	244	2.11	0.25	1.24	2.06	2.87	4.16
2017	17,121	369	2.14	0.18	1.21	2.07	2.94	4.31
2018	16,499	305	2.14	0.2	1.19	2.05	2.95	4.39
2019	15,871	300	2.2	0.2	1.23	2.1	3.03	4.53
2020	15,522	296	2.39	0.23	1.36	2.26	3.26	4.95

### 3.4. Consumption by pharmaceutical formulation in DDDANAT

**Figure 3:**
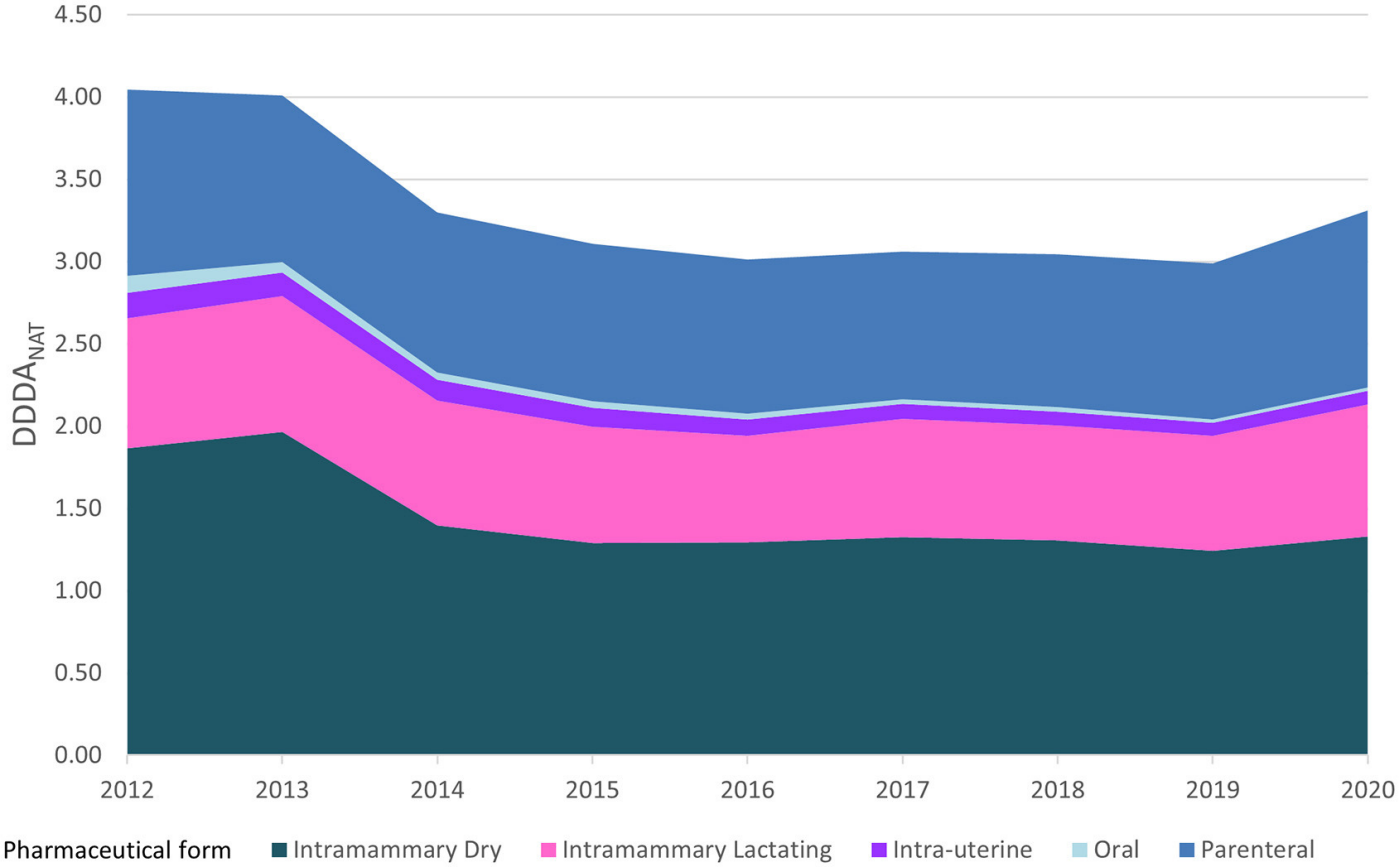
Antimicrobial consumption in the Dutch dairy sector, in DDDA_NAT_ units, on a yearly basis from 2012 to 2020, segmented into the different pharmaceutical formulations and the respective weight proportion in each year.

Figure 3 represents the evolution of the sectorial AMC in DDDA_NAT_ along with the relative weight of the consumption of different pharmaceutical formulations, from 2012 to 2020. Intramammary forms, both for lactating and dry cows, constituted more than 60% of the annual consumption over this period. However, the relative weight of intramammary formulations for the dry period in the yearly consumption dropped from 46 to 40% and the intramammary treatments for cows in lactation remained constant in DDDA_NAT_ units, but increased relatively from nearly 20–24%, due to the overall reduction registered. The consumption of intramammary formulations for dry cows dropped from 2013 to 2014 after the introduction of the guideline for selective dry-cow treatment, and after this marked reduction, it stayed roughly constant until 2020. The overall consumption of lactating intramammary formulations stayed approximately the same, with slightly lower DDDA_NAT_ levels between 2015 and 2019, but increased again in 2020. Oral treatments represent only a small part of the overall usage weighing about 2.7% in 2012 and 0.6% of the treatments in 2020 and intra-uterine formulations decreased from 3.7 to 2.4%. The usage of parenteral AM has remained approximately constant over the years, in number of DDDA_NAT_, but the relative weight increased from 28 to 32%.

### 3.5. Dynamics of combined pharmaceutical formulation and national line of choice

Table 2 breaks down the consumption of antimicrobial substances by both line of choice and pharmaceutical formulation and shows trends in AMC over the 2012–2020 period. Overall, during the 9 years covered, there was a reduction of 18% in the consumption of all pharmaceutical forms (Figure 2), with a reduced consumption of most pharmaceutical formulations, except for intramammary treatments for cows in lactation that remained almost constant, with only a 2% change from 2012 to 2020, and showing a percentual increase from 20 to 24% of the overall treatments, in Figure 3. Parenteral overall usage was reduced by 5%, followed by reduction in intramammary formulations for the dry period of 29%. Oral and intrauterine consumption showed marked reductions of 78 and 47% respectively.

Table 2 presents the calculation of annual variations in AMC, allowing us to dissect the sudden rise from 2019 to 2020. Firstly, as already identified in Figure 2, it shows that all AM products showed an increase from 2019 to 2020, higher for first line products (11%), and (8%) in second line formulations. Additionally, it can be seen that parenteral, intramammary lactation and intramammary dry forms are mainly responsible, in absolute terms, for the increase, respectively 0.12, 0.1, and 0.09 DDDA_NAT_. The relative changes were 13.3% for parenteral, 14.7% for lactating IM and 7.1% for dry cow IM formulations.

The reduction in second line AMC might be partially attributed to a shift in the consumption of both intramammary formulations to first line. After a 3-year reduction in AMC of second line parenteral formulations, there was an increase from 2016 to 2020 of 0.045 DDDA_NAT_, however, over the studied period AMC still decreased by 64% from 0.351 DDDA_NAT_ to 0.128 DDDA_NAT_.

Regarding first line products, the use of dry cow formulations increased in 2013 to 1.406 DDDA_NAT_ (44%) after which, it remained at around 1.2–1.4 DDDA_NAT_. In 2017, first line intramammary antimicrobials for use during lactation were introduced in the market.

The use of first line parenteral products first rose in 2013 (19%), then it remained steady until 2019 and it grew 12% in 2020.

Oral and intrauterine formulations consumption was markedly reduced for both first and second line products. The use of third line products was below 0.01 DDDA_NAT_ from 2013 onwards and only shows apparently arbitrary fluctuations.

### 3.6. Consumption of antimicrobial active ingredients by pharmaceutical formulation and national line of choice

**Figure 4:**
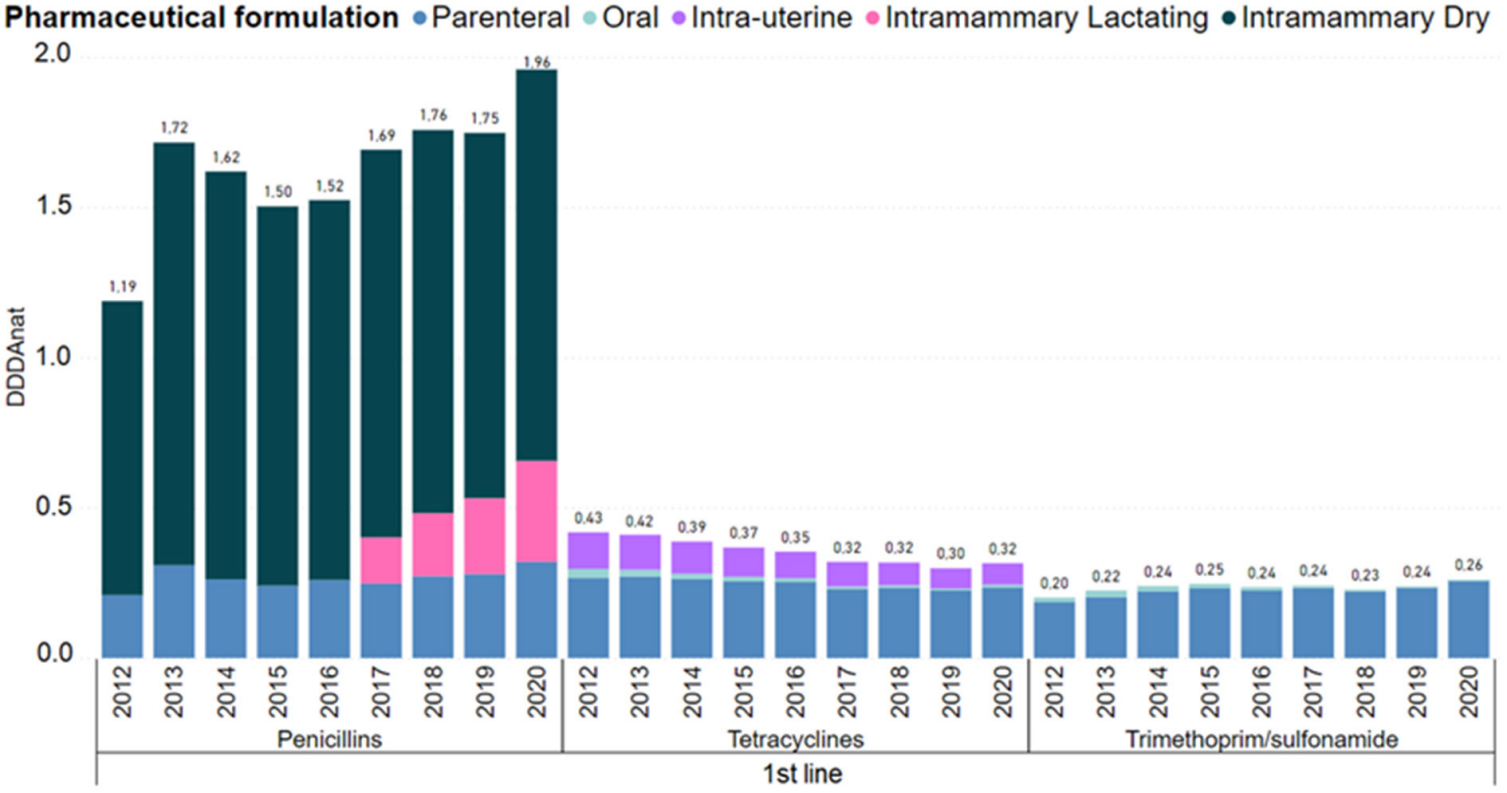
Antimicrobial consumption in the Dutch dairy sector of 1st line products, in DDDANAT units, from 2012 to 2020, segmented into the different pharmaceutical formulation. Amphenicols, macrolides/lincosamides were left out because each group represented <5% of the overall use in this line of choice. Full graph can be consulted in the Supplementary material.

In Figure 4, the consumption of first line molecules is detailed by pharmaceutical formulation. Penicillins usage grew substantially from 2012 to 2013, decreased from 2013 to 2015, then from 2015 to 2016 grew slightly, and from 2017 to 2020, with the introduction of intramammaries for lactating cows, it increased significantly. In penicillins, the parenteral use remained stable as well as the intramammary dry-cow therapy.

The use of trimethoprim/sulphonamides increased 30% from 2012 to 2020, and the use of tetracyclines, dominantly in the parenteral and intrauterine forms was reduced 23%. The usage of other first line products remained roughly constant and are not presented in the Figure 4, because these molecules represent <5% of the use in this line of choice.

**Figure 5:**
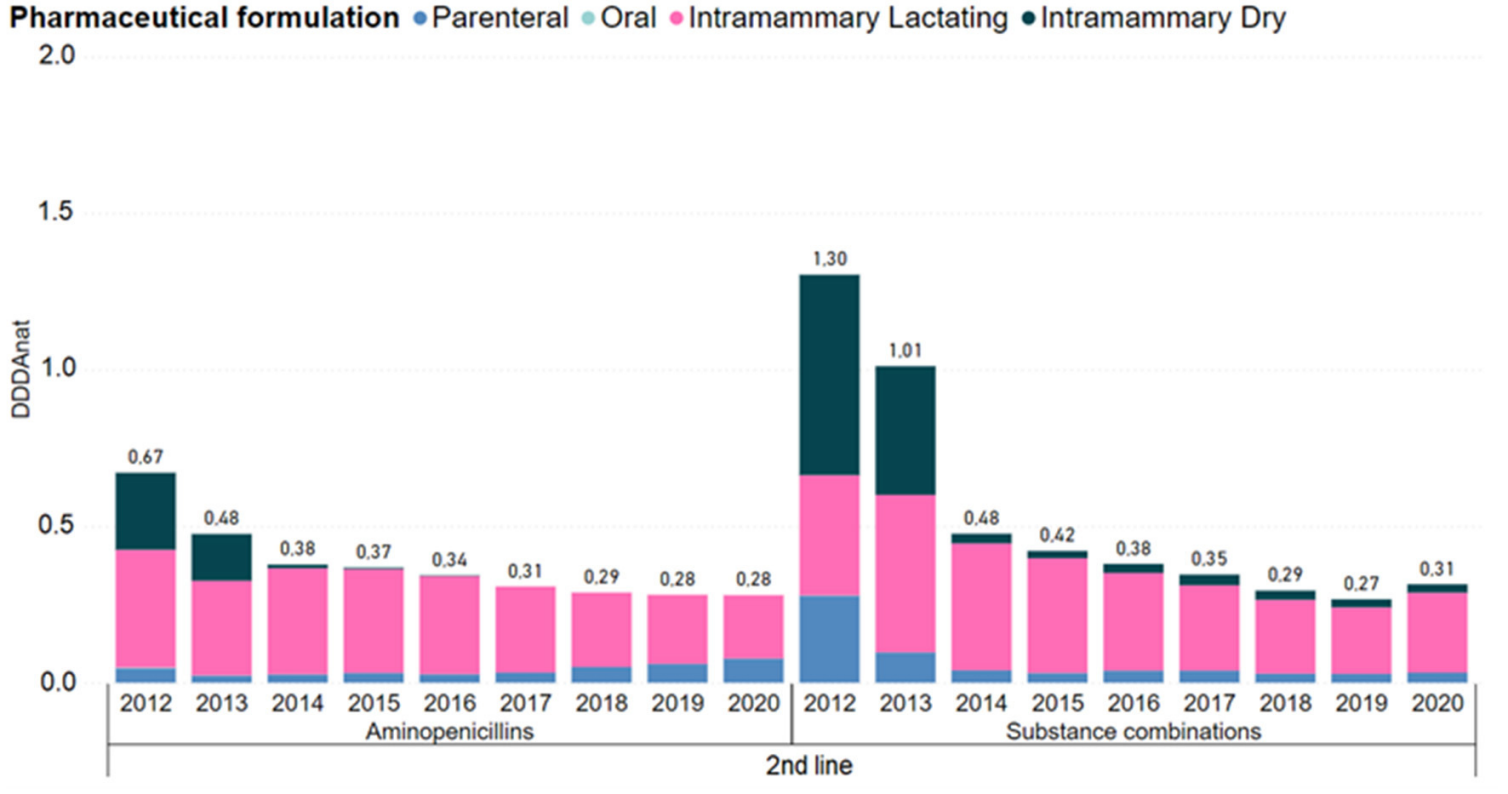
Antimicrobial consumption in the Dutch dairy sector of 2nd line products, in DDDA_NAT_ units, from 2012 to 2020, segmented into the different pharmaceutical formulations. Aminoglycosides, cephalosporins 1st and 2nd gen, long-acting macrolides, polymyxins and quinolones were left out because each group represented <5% of the use in this line of choice. Full graph can be consulted in the Supplementary material.

Second line products showed an overall decrease of 70% in usage. Figure 5 details consumption by pharmaceutical formulation and molecule of this category. The usage of aminopenicillins and substance combinations remained relevant, despite following the same decreasing trend. Regarding aminopenicillins, use of intramammary forms for dry cows disappeared in 2015 and lactation IM forms although reduced, remained with a considerable level of usage. The use of parenteral substance combinations was also reduced to very low consumption levels.

### 3.7. Use of critical molecules

Even though the use of third line products was already very low, in 2012, around 0.058 DDDA_NAT_, Table 2, over the last 9 years, there was a substantial reduction in the use of critical molecules such as cephalosporins of 3rd and 4th generation, fluoroquinolones and polymyxins. There is an absolute reduction in the proportion of farms using these groups of molecules, as presented in Table 4. Currently almost no farms are using cephalosporins of 3rd and 4th generation or polymyxins and only about 6% of farms remain attached to the use of fluoroquinolones. However, the proportion of farms using fluoroquinolones remains approximately stable since 2013.

**Table 4:** Number of farms that consumed 3rd line products and polymyxins, from 2012 to 2020, their yearly variation and percentage in the whole sector.

**Year**	**3rd and 4th gen Cephalosporines**	**%Total farms**	**Fluoroquinolones**	**%Total farms**	**Polymyxins**	**%Total farms**
2012	2,838	16%	2,554	14%	4,474	25%
2013	606	3%	1,335	7%	2,252	13%
2014	327	2%	1,244	7%	1,192	7%
2015	332	2%	1,321	7%	871	5%
2016	273	2%	1,238	7%	698	4%
2017	201	1%	899	5%	354	2%
2018	177	1%	900	5%	349	2%
2019	139	1%	898	6%	301	2%
2020	34	0%	945	6%	308	2%

## 4. Discussion

### 4.1. Validation of Vet-AMNet system

By accurately recreating the Dutch indicators and achieving the same figures as previously generated and published by the SDa that were compiled in this analysis, the Vet-AMNet system was successfully validated, demonstrating robustness to manage and relate nation wide antimicrobial prescription data with the respective drug characteristics and animal registry datasets, as will be demanded to produce national reports. The system's flexibility was evidenced by the adaptation of the original Vet-AMNet data model to include only the necessary information to allow the calculation of Dutch specific AMC indicators (DDDA_NAT_ and DDDA_F_). The original Vet-AMNet data architecture also includes information related to AM costs, milk yield values or field questionnaires, because it is intended to also be used as an analysis tool to assist farmers and veterinarians in AMC related decision making. These parameters were not included in this analysis because in the Netherlands, communication with individual farmers and veterinarians is the livestock sector's responsibility. Scientific and technological advancements together with new health and societal challenges may justify changes in a surveillance system. So, it is relevant to frequently evaluate the system's performance in meeting the proposed objectives, while operating under a budget (6). Other critical success factors will need to be evaluated by the Vet-AMNet management team once it is fully implemented. These are, among others, the user friendliness and acceptability of the system, cost efficiency, and the safety and quality of the data according to the FAIR (findability, accessibility, interoperability, and reusability) principle.

Microsoft Power BI^®^, the software of choice, is very versatile in what concerns data sources: it can be connected to Excel^®^,.csv files and SQL servers, among many others (7), providing the necessary universal compatibility mentioned which was very relevant for the implementation of the system in Portugal, given the fact that the partner dairy cooperatives, veterinary authorities and European institutions store data relevant for processing in a non-harmonized way. However, there is a 1 gigabyte limit to the data sets imported using the free version of the software (10).

Microsoft Power BI^®^ allows the creation and automatic update of dashboards and visuals, in real time, such as the graphs and tables presented in this paper. Even though the software also supports the creation of data visualizations using R and Python language, these can be built using the native Microsoft Power BI^®^ reporting interface in an intuitive drag and drop process that does not require programming skills to produce, once the relevant variables are set. This ease of use was very relevant to the initial stakeholder engagement process in Portugal. This makes the tool user-friendly for a broad scope of users and facilitates the customization of outputs. The SDa's expert panel found the reporting component of the Vet-AMNet system intuitive and a good tool to have in live discussions, where there is a need to quickly produce exploratory graphical outputs and tables.

The Vet-AMNet was developed to process data from the Portuguese dairy sector and used to analyze data from the Dutch dairy sector. However, the system's data architecture and data pre-processing procedures should be easy to adapt to other animal species and countries, providing that animal population data, antimicrobial sales and a national antimicrobial registry database are organized in a similar structure to the scheme in Figure 1 and there are interoperable codes that allow the establishment of connections between the different information sources.

During this work, several differences between the Portuguese and the Dutch systems were identified. To make a comparison between the two systems was not an objective of this paper, given that they have different overall aims. The Dutch system is mainly focused on producing annual reports, detailed information to transmit to the animal sectors and is a basis for the development of national antimicrobial stewardship measures. The aim of the Portuguese system is the communication of results to different actors, with data visualizations tailored to their needs. This highlighted the need to develop and conduct a structured and detailed framework analysis of the different systems in place to report national veterinary AMC information, to identify the best practices in the design and management of such systems to serve as a starting guide for newly developing countries and identify further possible improvements in already established ones.

### 4.2. Consumption of antimicrobials at sectoral level

The 9 years analysis of the AMC reveals an overall reduction of 18% in the use of AM in the dairy Dutch sector. Antimicrobial usage in the dairy sector is considered to be low and acceptable by the SDa, with narrow average DDDA_F_ distributions and only minor differences being observed between individual farms. AMC in the sector was stable from 2014 to 2019. From 2019 to 2020, although there was an 11% increase in the average consumption, in absolute terms it increased only around 0.3 DDDA_NAT_.

The decrease in consumption was pronounced in the first years after the implementation of the AMC monitoring and benchmarking system and stabilized after. From 2012 onwards the AMC of all dairy cattle farms was recorded, and benchmark values were set by the SDa. A signaling value of 3 DDDA_F_ and an action value of 6 DDDA_F_ were set originally (12). These values are subject to adjustments according to changes in the distribution of the DDDA_F_ of the dairy cattle farms. A farm that exceeds the action value needs to take immediate action to reduce their AMC. Farms with a usage level above the signaling value, but below action value require additional attention to reduce their usage, but no immediate measures must be taken. As the registered AMC of most dairy cattle farms at the start of the monitoring was considered relatively low, benchmark values aimed primarily at reducing the use of the persistently high users. When analyzing the percentile distribution of AMC registered by each farm, in DDDA_F_ units, it can be seen that more than 95% of the farms stayed below the action value during the whole period and around 75% stayed below the signaling value from 2014 to 2019, reflecting a consistent and sustainable level of control of the sectors consumption at farm level.

The introduction of more strict legislation allowing only first-line AMs for individual treatment to be available in small amounts for the farmer is likely connected with a shift of almost 15% in dry cow treatment from second line to first line products in 2013 (Figures 4, 5). Also, in 2017, the guideline for selective dry-cow treatment was introduced (8). The combination of these two measures, likely resulted in a 20% reduction of dry cow treatments, from 50 to 40% of total treatments (Figure 3). With the introduction on the market of first choice AMs for lactating cows in 2017, an increase in the overall national consumption of this pharmaceutical form is noted. In Table 2, the specific changes in lactating cow treatment are shown. In contrast to the dry-cow treatment, where a shift from second line to first line was noted, it looks like the introduction of first line injectors in 2017 (+0.151 DDDA_NAT_) only resulted in a small reduction in second line products (−0.078 DDDA_NAT_) resulting in an overall increase in 2017. In 2020, first line lactating cow injectors accounted for 0.336 DDDA_NAT_ (from 0.003 DDDA_NAT_ in 2016), while the second line lactating injectors accounted 0.467 DDDA_NAT_ (coming from 0.642 DDDA_NAT_ in 2016); an overall increase with 0.158 DDDA_NAT_. One of the confounding factors is the authorization of the new products. Products with cloxacillin are authorized for a treatment of 6 days, while the older second line products are authorized for a treatment of 1.5 days. When applied in concordance with the authorization, substitution of a second line treatment with the newly introduced first line treatment, might increase the number of DDDA with a factor 4. It is known from practice that the older products were sometimes applied for longer periods, and the new products won't be applied for the 6 days in all cases, so the impact of the new introduction won't be a factor 4 but might account for some increase in total consumption. When expressed in defined course doses (DCD's), which correspond to standardized units to represent a full course treatment, one would probably not even notice a decrease, if it is the case that DCD's are defined accordingly to the authorization (so 1 DCD for the older products would be 1.5 DDD, and 1 DCD for the new product would be equivalent with 6 DDD).

The registered AMC changes in the years 2017–2020, with overall values being stable from 2017 to 2019 and slightly increasing from 2019 to 2020, is for more than 50% attributable to the shift of second line lactating cow injectors to first choice ones. Additionally, between 2019 and 2020, an increase in parenteral treatments of 0.126 DDDA_NAT_, with 80% of this increase being first line AM's, is noticed and can't be explained by a change of products. However, given the low absolute usage levels no immediate action is required (9).

### 4.3. Consumption categorized by recommendation of 1st, 2nd or 3rd line

A strong shift from second line to first line AM products was shown in the first years, from 2012 to 2014, mostly connected with the intramammary treatments for dry cows, and this tendency was kept in subsequent years. Third line AM products do not play a relevant role in the sector, given the overall negligible level of consumption.

## 5. Conclusions

The Vet-AMNet system demonstrated to be sufficiently robust to encompass a country-wide AM monitoring program and to meet the critical success factors identified. Starting in 2023, it is projected that it will be used by the Portuguese veterinary authority to analyze national AMC trends and on a sample number of farms it will be implemented as a decision tool, where AMC figures will be presented with other relevant data. The antimicrobial stewardship initiatives adopted in the Netherlands demonstrated to be successful in the Dutch dairy sector, given the sector's low and acceptable AMC.

## Data availability statement

The data analyzed in this study is subject to the following licenses/restrictions: Dutch dairy sector farmers own the data. Results can be consulted in the yearly SDa reports. Requests to access these datasets should be directed to sanders@autoriteitdiergeneesmiddelen.nl.

## Ethics statement

Ethical review and approval was not required for the animal study because we analyzed medicines sales data. All the treatments were independent from the study. Written informed consent was obtained from the owners for the participation of their animals in this study.

## Author contributions

PM and JN-R designed the original system used for the data analysis. PM, PS, and IVG adapted the original system to process Dutch data. PM and IVG crafted the original study that was later expanded by PM and JN-R. All authors read and commented on the manuscript and agreed on the final version.
