# Supplementary material for: Use of a new antimicrobial consumption monitoring system (Vet-AMNet): Application to Dutch dairy sector over a 9-year period
Source: Front Vet Sci. 2022 Dec 15;9:984771. doi: 10.3389/fvets.2022.984771 (PMC9797842; doi:10.3389/fvets.2022.984771)
Supplement: Supplementary file 1 [file Data_Sheet_1.docx]

Supplementary table 1: Pharmacotherapeutic groups of the medicines registered in the Dutch “DG-standard” and their respective line of use.

Supplementary Material

| Line of choice | Pharmacotherapeutic group |
| --- | --- |
| 1st | Amphenicols |
| 1st | Macrolides/Lincosamides |
| 1st | Penicillins |
| 1st | Tetracyclines |
| 1st | Trimethoprim/sulphonamides |
| 2nd | Aminoglycosides |
| 2nd | Aminopenicillins |
| 2nd | Cephalosporins 1st and 2nd gen |
| 2nd | Quinolones |
| 2nd | Substance combinations |
| 2nd | Long-acting Macrolides |
| 2nd | Polymyxins |
| 3rd | Cephalosporins 3rd en 4th gen |
| 3rd | Fluoroquinolones |

$$\boldsymbol{DDDA}_{\boldsymbol{NAT}}\boldsymbol{=}\frac{\Sigma treated {kg}_{i, j}\times{amount}_{i, j}}{{number of animals}_{j}\times{std weight}_{j}} \boldsymbol{Formula} \boldsymbol{1}$$

Treated kg _i, j_ : the number of treated kilograms of a single package of antimicrobial i in livestock sector j
Amount _i, j_ : the number of packages prescribed of antimicrobial i in livestock sector j
Number of animals _j_: the number of animals present on average in livestock sector j
Std weight _j_: the standardized weight of an animal in livestock sector j

$$\boldsymbol{DDDA}_{\boldsymbol{F}}\boldsymbol{=}\frac{\Sigma treated {kg}_{i, j}\times{amount}_{i, j}}{{number of animals}_{j}\times{std weight}_{j}}\boldsymbol{Formula} \boldsymbol{2}$$

Treated kg _i, j_ : the number of treated kilograms of a single package of antimicrobial i on farm j
Amount _i, j_ : the number of packages prescribed of antimicrobial i on farm j
Number of animals _j_: the number of animals present on average on farm j
Std weight _j_: the standardized weight of an animal in livestock sector j

**Supplementary table 2: Standardized weight classes used for calculating the indicators DDDA_NAT_ and DDDA_F_, adapted from the standard operating procedure of the SDa** (Netherlands Veterinary Medicines Institute (SDa), 2020)**.**

| Indicator | Animal class | Age group | Standard weight in kilograms |
| --- | --- | --- | --- |
| DDDA_NAT_ | Dairy cattle | general | 600 |
| DDDA_F_ | Dairy cattle | >2 years | 600 |
|  | Heifers | 1-2 years | 440 |
|  | Yearlings | 56 days – 1 year | 235 |
|  | Calves (female) | <56 days | 56.5 |

2012 2013 2014 2015 2016 2017 2018 2019 2020

Supplementary figure 1: Antimicrobial consumption in the Dutch dairy sector in DDDA_F_ units, representing percentile 5, 25, 50, 75 and 95

Supplementary figure 1: Antimicrobial consumption in the Dutch dairy sector of 1st line products, in DDDANAT units, from 2012 to 2020, segmented into the different pharmaceutical formulation. Amphenicols, macrolides/lincosamides were left out because each group represented less than 5% of the use in this line of choice. Full graph.


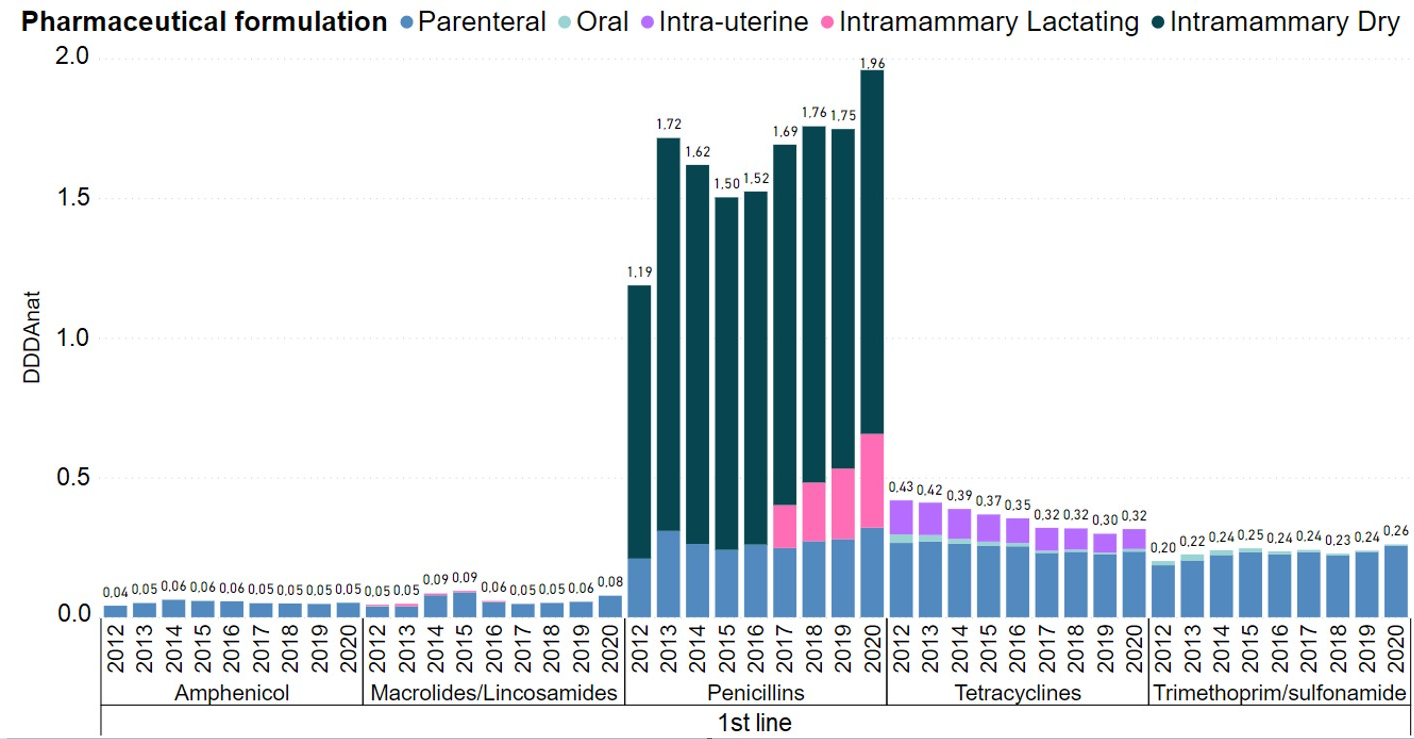


Supplementary figure 2: Antimicrobial consumption in the Dutch dairy sector of 2^nd^ line products, in DDDA_NAT_ units, from 2012 to 2020, segmented into the different pharmaceutical formulations. Aminoglycosides, cephalosporins 1st and 2nd gen, long-acting Macrolides, Polymyxins and quinolones were left out because each group represented less than 5% of the use in this line of choice. Full graph.


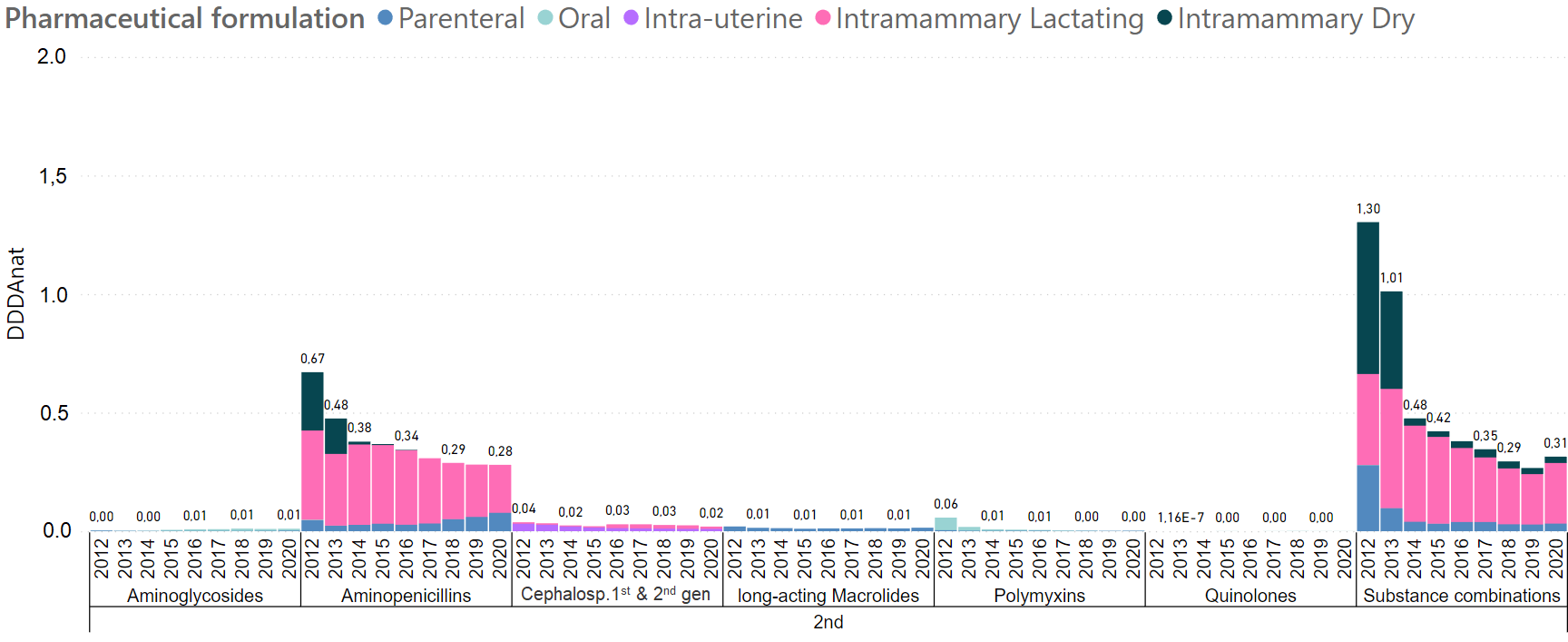


Supplementary table 3: Antimicrobial consumption in the Dutch dairy sector in DDDA_NAT_ units, from 2012 to 2020, segmented into the different pharmacotherapeutic group and lines of choice, adapted from (Netherlands Veterinary Medicines Institute (SDa), 2017, 2021a)

| Year | 2012 | 2013 | 2014 | 2015 | 2016 | 2017 | 2018 | 2019 | | 2020 | |  |
| --- | --- | --- | --- | --- | --- | --- | --- | --- | --- | --- | --- | --- |
| 1st line antibiotics | 1.91 | 2.47 | 2.39 | 2.27 | 2.23 | 2.35 | 2.4 | 2.39 | | 2.66 | |  |
| As a proportion of overall AB use | 47.06% | 61.23% | 72.56% | 73.06% | 74.00% | 76.90% | 79.00% | 79.90% | | 80.50% | |  |
| Amphenicols | 0.04 | 0.05 | 0.06 | 0.06 | 0.06 | 0.05 | 0.05 | 0.05 | | 0.05 | |  |
| Macrolides/lincosamides | 0.05 | 0.05 | 0.09 | 0.09 | 0.06 | 0.05 | 0.05 | 0.06 | | 0.08 | |  |
| Other | * | * | * | * | * | * | * | * | | * | |  |
| Penicillins | 1.19 | 1.72 | 1.62 | 1.5 | 1.52 | 1.69 | 1.76 | 1.75 | | 1.96 | |  |
| Pleuromutilins | * | * | * | * | * | * | * | * | | * | |  |
| Tetracyclines | 0.43 | 0.42 | 0.39 | 0.37 | 0.35 | 0.32 | 0.32 | 0.3 | | 0.32 | |  |
| Trimethoprim/sulfonamides | 0.2 | 0.22 | 0.24 | 0.25 | 0.24 | 0.24 | 0.23 | 0.24 | | 0.26 | |  |
| 2nd line antibiotics | 2.09 | 1.55 | 0.9 | 0.83 | 0.77 | 0.7 | 0.63 | 0.59 | | 0.64 | |  |
| As a proportion of overall AB use | 51.52% | 38.60% | 27.30% | 26.79% | 25.70% | 22.80% | 20.80% | 19.90% | | 19.30% | |  |
| Aminoglycosides | 0 | 0 | 0 | 0.01 | 0.01 | 0.01 | 0.01 | 0.01 | | 0.01 | |  |
| 1st- and 2nd-gen. cephalosporins | 0.04 | 0.03 | 0.02 | 0.02 | 0.03 | 0.03 | 0.03 | 0.03 | | 0.02 | |  |
| Quinolones | 0 | 0 | 0 | 0 | 0 | 0 | 0 | 0 | | 0 | |  |
| Substance combinations | 1.3 | 1.01 | 0.48 | 0.42 | 0.38 | 0.34 | 0.29 | 0.27 | | 0.31 | |  |
| Macrolides/lincosamides | 0.02 | 0.01 | 0.01 | 0.01 | * | * | * | * | | * | |  |
| Aminopenicillins | 0.67 | 0.48 | 0.38 | 0.37 | 0.34 | 0.31 | 0.29 | 0.28 | | 0.28 | |  |
| Polymyxins | 0.06 | 0.02 | 0.01 | 0.01 | 0.01 | 0 | 0 | Considered 3rd line | | | |  |
| 3rd line antibiotics | 0.06 | 0.01 | 0 | 0 | 0.01 | 0.01 | 0.01 | 0.01 | | 0.01 | |  |
| As a proportion of overall AB use | 1.42% | 0.18% | 0.14% | 0.15% | 0.30% | 0.20% | 0.20% | 0.20% | | 0.20% | |  |
| 3rd- and 4th-gen. cephalosporins | 0.04 | 0 | 0 | 0 | 0 | 0 | 0 | 0 | | 0 | |  |
| Fluoroquinolones | 0.01 | 0 | 0 | 0 | 0 | 0 | 0 | 0 | | 0 | |  |
| Polymyxins | Considered 2nd line | | | | | | | | 0 | | 0 | |
| Overall antibiotic use | 4.06 | 4.03 | 3.3 | 3.11 | 3.01 | 3.06 | 3.04 | 2.99 | | 3.31 | |  |

Supplementary table 4: Antimicrobial consumption and number of farms in the Dutch dairy in DDDA_F_ units, with Mean, Median, Percentile 75 and Percentile 90 figures from 2012 to 2020. Adapted from (Netherlands Veterinary Medicines Institute (SDa), 2021b)

| **Year** | **Mean** | **Median** | **P75** | **P90** |
| --- | --- | --- | --- | --- |
| 2012 | 2.9 | 2.72 | 3.75 | 5.6 |
| 2013 | 2.79 | 2.78 | 3.7 | 5.29 |
| 2014 | 2.27 | 2.19 | 3.04 | 4.47 |
| 2015 | 2.16 | 2.08 | 2.91 | 4.24 |
| 2016 | 2.11 | 2.06 | 2.87 | 4.16 |
| 2017 | 2.14 | 2.07 | 2.94 | 4.31 |
| 2018 | 2.14 | 2.05 | 2.95 | 4.39 |
| 2019 | 2.2 | 2.1 | 3.03 | 4.53 |
| 2020 | 2.39 | 2.26 | 3.26 | 4.95 |

References

Netherlands Veterinary Medicines Institute (SDa). (2017). *Usage of Antibiotics in Agricultural Livestock in the Netherlands in 2016*. *September*. https://www.autoriteitdiergeneesmiddelen.nl/en/news/21/sda-report-usage-of-antibiotics-in-agricultural-livestock-in-the-netherlands-in-2016

Netherlands Veterinary Medicines Institute (SDa). (2020). Standard operating procedure. In *Standard operating procedure*. https://doi.org/10.5055/jem.2005.0060

Netherlands Veterinary Medicines Institute (SDa). (2021a). *Appendix to the Report : Usage of Antibiotics in Agricultural Livestock in the Netherlands in 2020*.

Netherlands Veterinary Medicines Institute (SDa). (2021b). *Appendix to the Report :Usage of Antibiotics in Agricultural Livestock in the Netherlands in 2020*.
